# Predicting Generalized Anxiety Disorder From Impromptu Speech Transcripts Using Context-Aware Transformer-Based Neural Networks: Model Evaluation Study

**DOI:** 10.2196/44325

**Published:** 2023-03-28

**Authors:** Bazen Gashaw Teferra, Jonathan Rose

**Affiliations:** 1 The Edward S Rogers Sr Department of Electrical and Computer Engineering University of Toronto Toronto, ON Canada; 2 The Centre for Addiction and Mental Health Toronto, ON Canada

**Keywords:** mental health, generalized anxiety disorder, impromptu speech, linguistic features, anxiety prediction, neural networks, natural language processing, transformer models, mobile phone

## Abstract

**Background:**

The ability to automatically detect anxiety disorders from speech could be useful as a screening tool for an anxiety disorder. Prior studies have shown that individual words in textual transcripts of speech have an association with anxiety severity. Transformer-based neural networks are models that have been recently shown to have powerful predictive capabilities based on the context of more than one input word. Transformers detect linguistic patterns and can be separately trained to make specific predictions based on these patterns.

**Objective:**

This study aimed to determine whether a transformer-based language model can be used to screen for generalized anxiety disorder from impromptu speech transcripts.

**Methods:**

A total of 2000 participants provided an impromptu speech sample in response to a modified version of the Trier Social Stress Test (TSST). They also completed the Generalized Anxiety Disorder 7-item (GAD-7) scale. A transformer-based neural network model (pretrained on large textual corpora) was fine-tuned on the speech transcripts and the GAD-7 to predict whether a participant was above or below a screening threshold of the GAD-7. We reported the area under the receiver operating characteristic curve (AUROC) on the test data and compared the results with a baseline logistic regression model using the Linguistic Inquiry and Word Count (LIWC) features as input. Using the integrated gradient method to determine specific words that strongly affect the predictions, we inferred specific linguistic patterns that influence the predictions.

**Results:**

The baseline LIWC-based logistic regression model had an AUROC value of 0.58. The fine-tuned transformer model achieved an AUROC value of 0.64. Specific words that were often implicated in the predictions were also dependent on the context. For example, the first-person singular pronoun “I” influenced toward an anxious prediction 88% of the time and a nonanxious prediction 12% of the time, depending on the context. Silent pauses in speech, also often implicated in predictions, influenced toward an anxious prediction 20% of the time and a nonanxious prediction 80% of the time.

**Conclusions:**

There is evidence that a transformer-based neural network model has increased predictive power compared with the single word–based LIWC model. We also showed that the use of specific words in a specific context—a linguistic pattern—is part of the reason for the better prediction. This suggests that such transformer-based models could play a useful role in anxiety screening systems.

## Introduction

### Background

The screening, diagnosis, and tracking of mental health disorders require frequent interactions with psychiatrists or psychologists. However, the high cost [1] and low availability of mental health professionals make frequent interactions difficult [2]. This shortage could be addressed, in part, if there is an ability to assess a mental health disorder automatically through a passive and frequent collection of patient data. One possible way to do such monitoring may be through speech, as the presence of a mental health disorder has been shown to be associated with changes in human speech [3,4].

In this study, we focused on anxiety disorders, specifically on generalized anxiety disorder (GAD) [5]. Anxiety disorders are characterized by an excessive and uncontrollable fear of what is to come and are among the most common mental health issues, with an incidence of approximately 10% in the Canadian population [6]. It may be possible to reach a much greater proportion of the population using methods that automate some aspects of the measurement and diagnosis of anxiety disorders, such as the detection of anxiety from speech.

The current gold standard diagnosis for GAD requires multiple sessions with a mental health professional where the professional compares the different symptoms exhibited by the patient with the Diagnostic and Statistical Manual of Mental Disorders, Fifth Edition diagnostic criteria for GAD [7]. One place to look for symptoms is in the linguistic patterns used by the patient as the choice of words by anxious individuals tends to be different from that of nonanxious individuals [4]. The goal of this study was to determine the accuracy of a method for the automatic detection of anxiety from the transcript of impromptu speech. We were motivated to pursue this goal, in part, because it should be possible to frequently collect speech-to-text (STT) transcripts using smartphones or other wearable devices and, therefore, to enable a system for monitoring symptoms during or after treatment.

In recent years, transformer-based [8-12] neural network models [13] have been shown to have a strong capability to predict from language, including tasks such as next-word prediction, machine translation, and sequence classification. In this study, we leveraged this capability to predict whether a participant is above or below the screening threshold for GAD.

This paper is organized as follows: the *Prior Work* subsection summarizes related work in anxiety prediction from language and provides a brief overview of transformer language models. The *Methods* section describes the speech sample collection methods and the construction, training, and evaluation of the prediction model. The *Results* section presents the prediction model’s performance, whereas the *Discussion* section discusses specific patterns that were influential in the prediction.

### Prior Work

#### Previous Work on the Automatic Prediction of Anxiety From Speech

Several prior studies have explored the automatic prediction of anxiety from speech. These studies have used both the acoustic properties as well as the linguistic features of speech and have shown some ability to detect anxiety. Most prior studies have focused on the acoustic structure of speech, that is, the nature of the audio signal itself. Comparatively less work has been done on the linguistic aspects of speech, the focus of this paper, which we describe in the subsequent paragraphs.

Di Matteo et al [14] explored the relationship between passively collected audio data and anxiety and depression. A total of 84 participants installed an Android app on their smartphone for 2 weeks. During this period, the app passively collected intermittent samples of audio data from the participants’ smartphones. The audio was then converted to text, and the Linguistic Inquiry and Word Count (LIWC) [15] was used to classify the words into 67 different categories. The correlation between the LIWC scores and self-report measures was calculated for social anxiety disorder (SAD), GAD, depression, and functional impairment. A significant correlation was observed between words related to the perceptual process (“See” in the LIWC) and SAD (*r=*0.31; *P*=.003). In addition, words related to reward were significantly correlated with GAD (*r=*−0.29; *P*=.007).

Anderson et al [16] recruited 42 participants diagnosed with SAD and 27 healthy controls to explore the differences in the words used between these 2 groups using the LIWC features. An anxiety-stimulating task was performed in which the participants were asked to write about an autobiographical and socially painful memory, which required them to recall a social humiliation, embarrassment, or shame. The word count in each of the LIWC categories was generated, including first-person singular pronouns, anxiety-related words, and fear-related words. The patients with SAD used more first-person singular pronouns (I, me, and mine), anxiety-related words, sensory or perceptual words, and words denoting physical touch but made fewer references to other people than the healthy controls.

Hofmann et al [17] examined the association between linguistic features and SAD. They recruited 24 participants diagnosed with SAD and 21 healthy controls. The participants were asked to provide a speech on any topic of their choice for a total of 4 minutes in front of an experimenter while being video recorded. To induce stress and anxiety in the participants, they were told that a panel of judges would rate their speech after it was recorded on the basis of poise, social confidence, and general presentation skills. The speech was transcribed, and LIWC was used to extract the count of the words in the following categories: first-person pronouns, negative emotion words, and positive emotion words. The results showed that the patients with SAD used more positive emotion words than the healthy controls. The authors did not observe any significant difference for the other explored LIWC categories.

Sonnenschein et al [18] explored the transcripts from passively recorded therapy sessions of 85 patients. These patients were categorized into 3 groups: those diagnosed with anxiety but not depression, those diagnosed with depression but not anxiety, and those diagnosed with both anxiety and depression. From the transcripts, the LIWC score was generated in 4 categories: first-person singular, sad, anxiety, and filler. The group with depression but not anxiety showed a higher use of sad words than the group with anxiety but not depression. The group with anxiety but not depression showed a higher use of anxiety-related words than the group with depression but not anxiety. The *both anxious and depressed* group also showed a higher use of “sad” words than the group with anxiety but not depression. None of the other LIWC categories explored showed a significant difference.

Rook et al [19] attempted to predict GAD from linguistic patterns because they believed that the worrying behavior in GAD comes from the verbal linguistic process. A total of 142 undergraduate participants (n=56, 39.4% men and n=86, 60.6% women) were recruited for their study and were asked to recall and write about an anxious experience during their university life. The Generalized Anxiety Disorder 7-item (GAD-7) scale score and behavioral inhibition/behavioral approach system (BIS/BAS) scale score were used as the label for each of the participants. The LIWC features [15] were extracted from the texts written by the participants. Another set of features was also used by combining the LIWC features with the BIS/BAS scores. Several machine learning models were explored, including support vector machine (SVM) with linear kernel, logistic regression, naive Bayes, and random forest. Their results showed that all models built using the LIWC features performed significantly better than a random model (average precision~0.61; average recall~0.6) and achieved a higher performance (except for the SVM model) when the LIWC features were used together with the BIS/BAS scores as input features (average precision~0.65; average recall~0.64).

Gruda and Hasan [20] explored the prediction of anxiety from microblogs such as tweets using machine learning approaches. The authors started by labeling 600 tweets on a 4-point anxiety level using the short version of the traditional full-scale State-Trait Anxiety Inventory [21]. Then, a machine learning model was trained using features extracted from the textual content. The features used include a semantic embedding vector, which is the mean of multiple word vectors that map words to a vector. They also used the count of specific words and emojis as another type of features. They achieved an *R*^2^ of 0.49 between the human label and the predicted label after training a Bayesian ridge regression [22] model. The authors then compared their model with a model that classifies a tweet as anxious or not based on the presence of anxiety-type words and negative emotion–type words, which was acquired using the LIWC library. The method that used the LIWC features to classify between anxious and nonanxious tweets achieved an *R*^2^ of 0.21, indicating the importance of the meaning of words represented by word vectors.

A precursor study to this work [23] identified both acoustic features and linguistic features using LIWC that significantly correlated with the GAD-7. Using these features, in another study [24], a logistic regression model was trained to predict whether a participant was above or below the screening threshold for GAD based on the GAD-7. Using both the acoustic and linguistic features, we achieved a mean area under the receiver operating characteristic curve (AUROC) of 0.59.

Note that this previous study [24] and the other prior work, described in the previous paragraphs, explored the count of single words (using the LIWC) to find an association with anxiety or to predict anxiety. However, there are some studies that found specific word categories to be associated with anxiety, whereas others found no such association. For example, the studies by both Di Matteo et al [14] and Anderson et al [16] found that the word categories for “perceptual process” were associated with anxiety, whereas no other prior studies did so. Similarly, the first-person singular pronoun category was associated with anxiety only in the studies by Anderson et al [16] and Teferra et al [23] and nowhere else. These inconsistencies may be explained if the context for the specific words is taken into account—or, in other words, if the evaluation model is context aware. In this study, we hypothesized that there is a greater predictive power in examining the larger context of multiple words than in examining single words using LIWC. The former can be done using recent advances in natural language processing (NLP) [8], which has new powerful methods of converting language into numerical quantities that represent meaning and learning features that are patterns of those meanings.

Furthermore, note that the largest sample size among the previously explored studies (excluding our own [23,24]) was 142. This limits the potential for generalizability to a larger population. In this study, we used a much larger data set based on speech samples from a total of 2000 people.

#### Transformers and NLP

Over the last 5 years, substantial advances have been made in the field of NLP [25]. A key advance was the invention of limited-size word *vectors* or *embeddings*, through which it has been shown that a small-sized (from 50 to 300) vector of real numbers was capable of representing the meaning of individual words or parts of words [26-28]. Note that sometimes, words are divided into subparts and then converted into tokens, which can represent either a full or a partial word. These word or token vectors make it possible to determine whether 2 words have similar meaning through a numerical comparison, as well as other encapsulations of meaning through calculation. This invention also permitted the use of neural networks to process language in a far more effective way and has led to major advances in the subfields of speech recognition, natural language understanding, question answering, and language generation [26,29].

Another important step that has dramatically improved the state of the art in these fields is the advent of the transformer-based neural network models [8,10-12,30]. These so-called large language models are trained using massive corpora of text, often obtained from the internet. More specifically, the “learning” (in the machine learning sense [31]) is done by either predicting the next word in sequence or predicting intentionally missing words. The architecture of a transformer-style neural network has 2 important properties. First, it “transforms” a sequence of words or parts of words, represented as vectors, into another sequence of vectors. The output vectors account for additional meaning inferred from the full sequence of words and thus create a sequence of so-called contextual embeddings that better encapsulate the meaning of the full input sequence. Second, it makes use of an important neural network mechanism known as “attention” [8,32]. In this, a part of the network learns several different ways in which parts of a sentence or paragraph are related to other parts of the sentence. For example, a certain word or meaning may typically be connected with specific other words in a sentence. A transformer learns many such relationships, which makes it capable of classifying the broader meaning of a sentence or paragraph. It is this capability that we leverage in this study to look for patterns of language that indicate the presence of anxiety.

There now exist many such large language models that have already been fully “pretrained” on massive corpora of text gathered from a number of sources on the internet and elsewhere [10,12,30]. A common use case in the field of deep learning and NLP is to take such pretrained models and “fine-tune” them for a specific prediction task that takes language as input. To “fine-tune” a model means to train it on a (typically much smaller) data set to learn the task at hand. The task described in the subsequent section is the classification of participants into anxious or nonanxious categories.

## Methods

### Data Collection

#### Recruitment and Demographics

We note that this study used the same participants and data as 2 earlier studies [23,24]. This study performed a novel analysis of these data using a transformer-based neural network.

The participants were recruited using Prolific [33], a web-based human participant recruitment platform. The inclusion criteria were an age range of 18 to 65 years; fluency in English; English as a first language; and the completion of at least 10 previous studies on Prolific, with 95% of these previous Prolific tasks completed satisfactorily (as labeled by the study author). The data set was also balanced for sex (n=1000, 50% female and n=1000, 50% male).

The participants who completed the study were paid £2 (approximately CAD $3.41 and US $2.74) for approximately 15 minutes of work. They completed the entire study remotely using their PCs.

#### Ethics Approval

This study was approved by the University of Toronto Research Ethics Board (protocol #37584).

#### Study Procedure

The participants were recruited for a 10- to 15-minute task implemented through a custom website. An earlier study that determined the correlates of anxiety [23] described the data collection procedure in detail. The parts of the data collection procedure that are relevant for the purpose of this study are presented in the following paragraphs.

On the Prolific platform, the participants who met the inclusion criteria were presented with the opportunity to participate in this study. Those who wished to participate clicked on the study link, which brought them to a consent form that described the procedure and goals of the study and provided information on data privacy. If a participant granted consent, a hyperlink brought them to an external web application that implemented the tasks described subsequently.

The participants were asked to fill out the standard GAD-7 questionnaire [34], which is described in more detail in the *Anxiety Measures* section. Then, they were asked to perform a speech task, which was both audio and video recorded using their computer’s microphone and camera. The speech task followed a modified version of the widely used Trier Social Stress Test (TSST) [35], which aims to evoke a moderate amount of stress from each participant. Prior studies [36,37] have shown a higher activation in participants with relatively higher anxiety after they experienced moderate stress induced by the TSST.

In the modified version of the TSST, the participants were told to imagine that they were a job applicant invited for an interview with a hiring manager. They were told to imagine that it was a job that they really wanted—their so-called dream job. They were given a few minutes to prepare—to choose their dream job—and to think about how they would convince an interviewer that they were the right person for that position. The participants were also told that the recorded video would be viewed by researchers studying their behavior and language. The participants were then asked to speak for 5 minutes, making the case for themselves to be hired for that dream job.

Note that, in the original TSST [35], participants would normally deliver their speech in front of a live panel of judges. If a participant finished their delivery in <5 minutes, the judges in the original TSST design would encourage the participant to keep speaking for the full 5 minutes. For example, in the original TSST, to encourage the participants, they were asked the following question: “What are your personal strengths?” In the modified TSST, we implemented a similar method to encourage the participants to speak for the full 5 minutes: when our system detects silence (defined as the absence of speech for >6 seconds), it will display several different prompts inviting the participants to keep speaking on different topics relating to the task. Finally, note that the modified TSST only included the first task of the original TSST, not the second task, which involves the performance of mental arithmetic.

#### Anxiety Measures

Our goal was to predict, based on the transcript of the language spoken, whether a participant was above or below the screening threshold for GAD based on the GAD-7 scale. The GAD-7 [34] scale is a 7-item questionnaire that asks participants how often they were bothered by anxiety-related problems during the previous 2 weeks. Although the 2-week period suggests that the GAD-7 measures a temporary condition, a GAD diagnosis requires a 6-month duration of symptoms [7,38]. However, the GAD-7 has been validated as a diagnostic tool for GAD using a value of 10 as the cutoff threshold, with a sensitivity of 89% and a specificity of 82% [34]. Thus, we chose to use the GAD-7 threshold of 10 to obtain a binary label of GAD as our indicator of anxiety.

Each of the 7 questions on the GAD-7 has 4 options for the participant to select from, indicating how often they have been “bothered” by the 7 problems listed. These options and their numerical ratings are 0=not at all, 1=several days, 2=more than half the days, and 3=nearly every day. The final GAD-7 score is a summation of the values for all the questions, giving a severity measure for GAD in the range from 0 (no anxiety symptoms) to 21 (severe anxiety symptoms).

### Construction and Evaluation of the Baseline Classification Model

In this section, the inputs, structure, and evaluation of a baseline model are described. The inputs to this model were the linguistic features acquired using LIWC [15]. LIWC is based on the count of words from a given transcript that fall into different preset categories. An example category is “negemo,” which comprises words (such as hurt, ugly, and nasty) that are associated with negative emotion. The full set of categories in LIWC can be found in the study by Pennebaker et al [15].

The transcript was generated from the speech samples using Amazon Web Services STT system (Amazon.com, Inc) [39]—the transcription accuracy on a written text had an average word error rate (WER) of 7% (SD 4.6%). In our earlier study [23], we identified LIWC features that had a significant (*P*<.05) correlation with the GAD-7. These features are listed in Table 1. These were the features that were used as the input to the baseline prediction model.

A logistic regression model was trained to make predictions between the anxious and nonanxious classes. The construction and evaluation steps were as follows. First, the input features were normalized so that each feature would have a mean of 0 and an SD of 1. Next, the data were undersampled to equalize representation from both the anxious and nonanxious classes. This avoids the problem of class imbalance, which, if it occurs, causes low predictive accuracy for the minority class (which is the anxious class in our case). To undersample the data, samples were randomly selected and removed from the majority class until the majority class had an equal number of samples as the minority class.

The model construction and training steps used 3 data sets: a training data set (80% of the entire subsampled data), which was used to train the model; a validation data set (20% of the training data), which was used to select the best hyperparameters during training; and a test data set (20% of the entire subsampled data that were not included in the training data set), which was used to evaluate the performance of the trained model using the AUROC metric. This methodology—the careful separation of the training and validation data from the test data—is standard in the machine learning community [31].

**Table 1:** Correlation of significant Linguistic Inquiry and Word Count features with the Generalized Anxiety Disorder 7-item scale.

Feature	*r*	*P* value
AllPunc	0.13	<.001
Word Count	−0.12	<.001
Period	0.12	<.001
Assent	0.10	<.001
Negemo	0.10	<.001
Relativ	−0.09	<.001
Motion	−0.08	<.001
Swear	0.08	<.001
Anger	0.08	<.001
Focusfuture	−0.07	.003
Adverb	−0.07	.004
Time	−0.07	.004
Function	−0.07	.005
Negate	0.07	.006
Prep	−0.06	.007
WPS^a^	−0.06	.007
Anx	0.06	.008
Hear	0.06	.01
Death	0.06	.01
Ipron	−0.06	.01
See	−0.06	.01
Affect	0.06	.02
I	0.05	.02
Family	0.05	.02
Sad	0.05	.03
Ppron	0.05	.03
Space	−0.05	.04
Article	−0.05	.04
Leisure	0.05	.04
Friend	0.05	.047

^a^WPS: words per sentence.

### Construction and Evaluation of the Transformer-Based Model

The advent and remarkable success of transformer-based neural networks for NLP is discussed in the *Prior Work* section. A property that distinguishes different transformer models is the number of textual words or tokens that will fit into the contextual window that the model can consider at one time, which itself is limited by the computational burden of the key method of attention [8]. These windows range in size from 512 tokens [10] to 4096 tokens [30].

The modified TSST that provided the input to our model required the participants to speak for 5 minutes, which produced transcripts ranging in size from 15 to 1190 (mean 707, SD 183) tokens. Therefore, our model required a transformer model that can process sequences of this length. We selected the transformer model known as Longformer (obtained from the HuggingFace model hub [40]) because it has a contextual window of size 4096 tokens (recall that tokens are either words or parts of a word).

We *fine-tuned* a pretrained version of Longformer (as described in the *Prior Work* section) to create a classifier for the anxiety classification task. This process took a pretrained model and attached it to an untrained (and much smaller) neural network called a “classification head.” The pretrained model together with the sequence classification head was then *fine-tuned* on the specific task of predicting whether a participant is above or below the screening threshold for GAD based on the GAD-7 scale.

The input data set was processed in a similar way to how the baseline model was processed. Beginning with the set of transcripts from all the participants, the data were first undersampled to equalize the representation from both the anxious and nonanxious classes. The model fine-tuning step also used 3 data sets: a training data set (80% of the full data); a validation data set (20% of the training data); and a test data set (20% of the full data set), which was used to evaluate the performance of the trained model using the AUROC metric.

**Figure 1:**
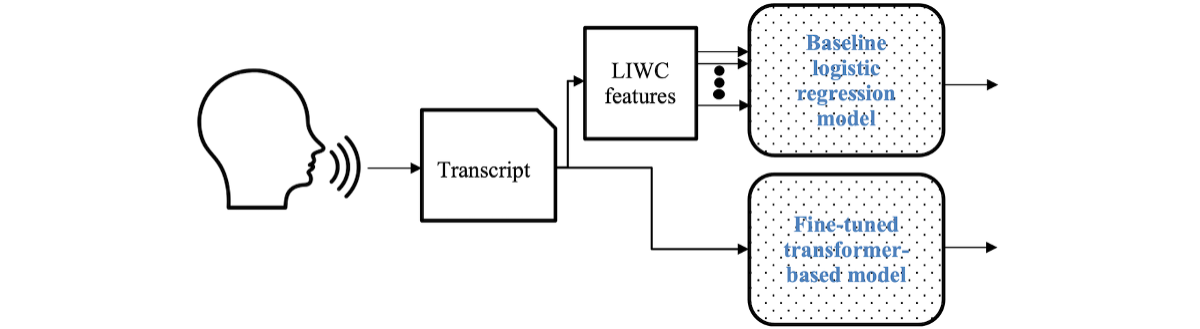
Overall structure of the baseline classification model and fine-tuned transformer-based model. LIWC: Linguistic Inquiry and Word Count.

The overall structure of both the baseline logistic regression model and the fine-tuned transformer-based model is shown in Figure 1.

### Transformer Model Interpretation

Deep neural networks [13], including the transformer network used in this study, do not lend themselves to an easy explanation of which features or factors are important for any specific prediction. This contrasts with the logistic regression model (the baseline) in which the weights on each feature are informative. This study endeavored to provide some interpretation of the results of the transformer model, particularly, to provide insights into which words or group of words were the most influential in the model’s prediction of anxious and nonanxious classes when given a specific transcript.

To achieve this model interpretation, we used a method known as integrated gradient (IG) [41]. IG computes a score for every input (word or token) to the model. The score is a function of the rate of change of the prediction with respect to that specific input. When the score of specific input is higher and positive, it is an indication that the input had more influence toward producing a positive classification (which is the anxious class in our case). Similarly, a high negative score indicates a strong influence toward the negative, nonanxious case. This score is referred to as the *attribution* score of the input token. We used a library called Transformer Interpret [42] to compute the attribution score for each word in a given transcript.

Using the attribution score, we can report specific words or tokens that are influential in the prediction of both anxious and nonanxious cases. From there, we explored the specific context of those words to look for patterns of language that were influential. The description in the following paragraphs provides the specific method for selecting words and identifying patterns.

**Figure 2:**
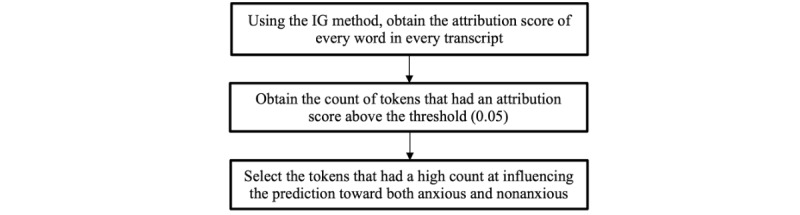
Steps to obtain the list of tokens with a high attribution score and high count at influencing the prediction toward both anxious and nonanxious. IG: integrated gradient.

First, the attribution score of each word or token in all the transcripts from all the participants was computed. In the plot of the distribution of the number of words with each score, the knee of the distribution appeared around a threshold attribution score of 0.05, which provided a tractable number of words to explore. The tokens with scores above the threshold of 0.05 are presented in the *Results* section. A summary of the steps we took to get the list of words is shown in Figure 2.

To determine whether there were patterns in the context surrounding the high-attribution words, we manually reviewed the surrounding context of each high-attribution word. The patterns we observed from these contexts, together with the specific direction of the prediction (anxious or nonanxious), are presented in the *Results* section.

## Results

### Recruitment and Data Inclusion

A total of 4542 participants accepted the offer from the Prolific recruitment platform to participate in this study. Of them, 2212 participants finished the study, giving a recruitment yield of 48.7%. Of the 2212 participants who completed the study, 2000 provided acceptable submissions (and thus received payment), giving a submission-to-approval yield of 90.4%. To be clear, the recruitment continued until 2000 acceptable submissions were received. The reasons for which submissions were deemed unacceptable include the following: a missing video, missing or grossly imperfect audio, and failure to complete the task. The recruitment period lasted from November 23, 2020, to May 28, 2021. We note that the recruitment was conducted during the COVID-19 pandemic.

### Data Overview

Of the 2000 participants, 620 (31%) were above the GAD-7 screening threshold of 10 and 1380 (69%) were below the screening threshold of 10. Henceforth, the participants with a GAD-7 score ≥10 are referred to as the anxious group, and those with a GAD-7 score <10 are referred to as the nonanxious group. As described in the *Methods* section, to have an equal representation of the anxious and nonanxious classes, the nonanxious group was undersampled, resulting in the inclusion of a total of 1240 participants (620 anxious and 620 nonanxious) in our analysis.

### Classification Model Performance

**Figure 3:**
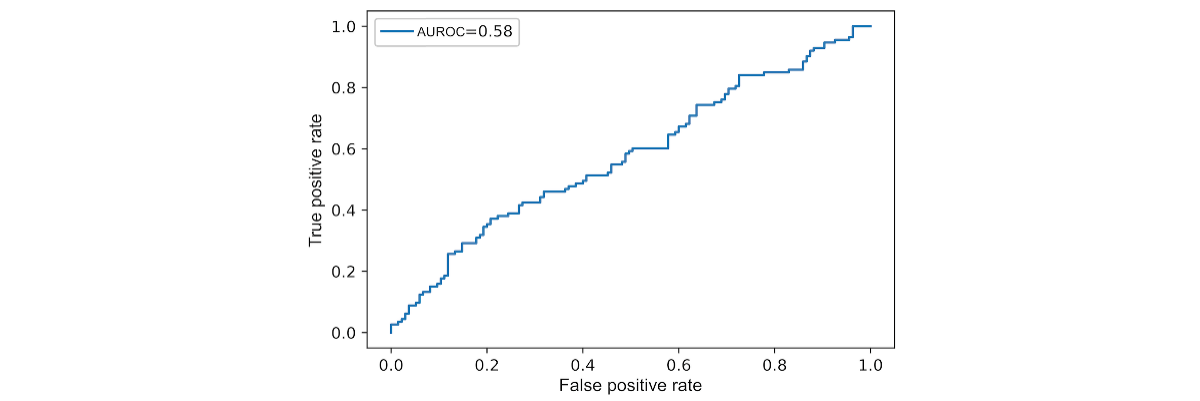
Area under the receiver operating characteristic curve (AUROC) of the baseline logistic regression model.

**Figure 4:**
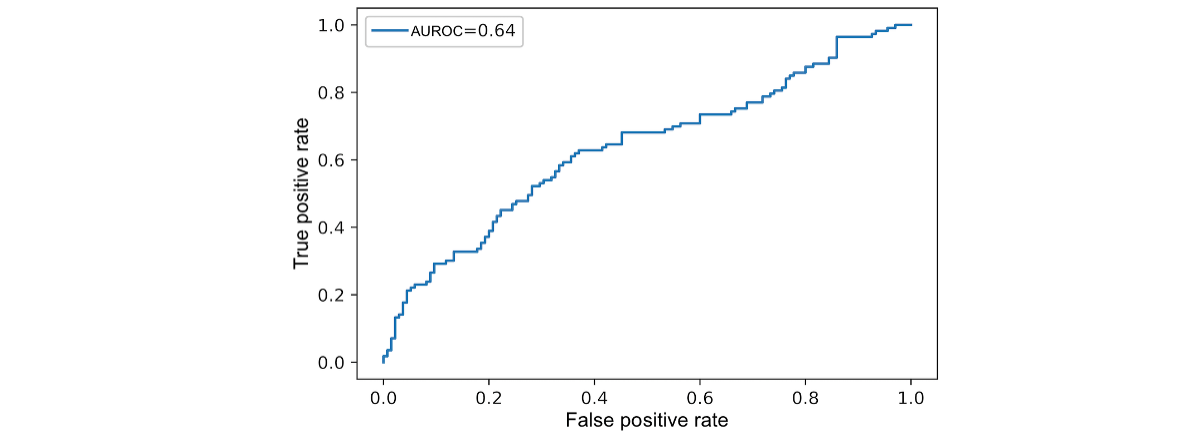
Area under the receiver operating characteristic curve (AUROC) of the fine-tuned transformer-based model.

This section presents the AUROC of the 2 binary classification models that classify anxious and nonanxious groups. The first model is the logistic regression model that uses the LIWC features as input, which is the baseline model described earlier. The LIWC features used were the ones shown to be significantly correlated with the GAD-7 in our earlier study [23], as listed in Table 1. Note that we also explored other machine learning models such as SVM, decision tree, random forest, multilayer perceptron, but these did not perform better than the baseline logistic regression model. The second model is the fine-tuned transformer-based model. The AUROC curve value for the logistic regression model that uses the LIWC features as input was 0.58 and for the transformer-based model was 0.64. Figures 3 and 4 present the receiver operating characteristics curves.

### Model Interpretation: Tokens Used to Predict Both Anxious and Nonanxious

In the *Transformer Model Interpretation* section, we described the IG method that was used to determine an attribution score for each word in a transcript. That score gives an indication of how strongly the word is implicated in the prediction toward anxious (if positive) or nonanxious (if negative). Table 2 presents the number of times (across all transcripts) that a specific token (listed in the first column) had a high attribution score (absolute value >0.05, as described earlier) based on the IG method. The tokens presented in Table 2 were selected because they had a high count in having both high positive and high negative attribution scores, that is, at predicting both anxious and nonanxious. Note that tokens could be words, parts of a word, or characters (eg, the STT system we used generates a “.” to indicate silent pauses in speech).

Table 3 presents the patterns we observed with examples taken from the actual transcripts of the recruited participants where the same token influenced the prediction toward anxious in some cases and toward nonanxious in other cases. The first column lists these tokens, indicates the direction (anxious or nonanxious) in which they influenced the prediction, and describes the pattern of the context that we inferred was relevant using the qualitative analysis described in the *Methods* section. The second column provides a specific example of that pattern, taken from the transcripts, and the third column provides the number of occurrences of that pattern across all the transcripts.

**Table 2:** Tokens with high attribution scores and high counts of prediction influence.

Token	Times influencing toward anxious, n (%)	Times influencing toward nonanxious, n (%)
I (n=3459)	3032 (87.65)	427 (12.35)
[Silent pause]^a^ (n=14,490)	2933 (20.24)	11,557 (79.76)
[Filled pause]^b^ (n=3434)	2039 (59.38)	1395 (40.62)
And (n=1595)	913 (57.24)	682 (42.76)

^a^[Silent pause]: a silent pause in speech, as determined by the speech-to-text software.

^b^[Filled pause]: a pause consisting of filler words such as “um,” “mm,” “uh,” “hmm,” or “mhm.”

**Table 3:** Cases in which the tokens in influenced the prediction of both anxious and nonanxious.

Token, prediction class, and definition of pattern			Example of pattern	Occurrences across all transcripts, n
**I**				
	**Anxious**			
		“I” followed by a filled pause^a^	I have um I worked very well	476
		“I” is the first word in a sentence but in the middle of the transcript	I get on well with various different groups	1567
		Starting a sentence and pausing after just saying “I”	I [Silent pause]	208
		“I” used together with am or have	I am able to relate	1515
	**Nonanxious**			
		“I” used in a sentence to reference others	I was able to remember all their names	47
		“I” is the very first word in the transcript	<speech starts> I think I would be perfect for this job	171
		“I” used to describe a positive thing about oneself	I am imaginative	77
**[Silent pause]^b^**				
	**Anxious**			
		[Silent pause] used before or after a [Filled pause]	[Silent pause] um mm [Silent pause]	1740
		Starting a sentence and pausing within a short period	my [Silent pause]	2057
	**Nonanxious**			
		Pauses during speech that are not accompanied by a [Filled pause] and produce a correct sentence	bring a specific [Silent pause] area of expertise of functionality	11,557
**[Filled pause]^a^**				
	**Anxious**			
		[Filled pause] used together with a [Silent pause]	[Silent pause] um mm [Silent pause]	1577
		[Filled pause] used in the beginning of a speech	<speech starts> hello um I just like to	23
	**Nonanxious**			
		Filled pause used in the middle of a sentence without a silent pause	many years playing music at parties *um* starting at the age of	480
**And**				
	**Anxious**			
		Finishing a sentence with “and”	really think about it in detail and	519
		Using “and” more than once in a sentence	was tasked in doing that and and I did that successfully and that	187
		Starting a sentence and pausing after just saying “and”	and [Silent pause] sometimes things are	282
	**Nonanxious**			
		“and” used grammatically correctly in a sentence	eight people for twelve years and after that I managed an additional	572

^a^[Filled pause]: a pause consisting of filler words such as “um,” “mm,” “uh,” “hmm,” or “mhm.”

^b^[Silent pause]: a silent pause in speech, as determined by the speech-to-text software.

## Discussion

The goal of this study was to determine how well a transformer-based neural network model can predict GAD and compare it to the performance of an LIWC-based logistic regression predictor. In this section, we discuss the implications of the findings presented in the *Results* section, as well as the limitations of the study.

### Principal Findings

#### Recruitment and Data Overview

Results presented in the *Data Overview* section indicates that a substantially larger number of participants screened positive for GAD compared with the prevalence rate of 10% in the general population [6]. This suggests that participants recruited from Prolific are more likely to experience anxiety, which is consistent with previous research using participants from Prolific [23,43,44]. Another possible reason for a higher number of anxious participants is the recruitment period (November 23, 2020, to May 28, 2021), which coincided with the COVID-19 pandemic. More demographic information can be found in our earlier published papers [23,24].

#### Classification Model Performance

The logistic regression model with LIWC features is the baseline point of comparison. This model performed better than the random model (as it has an AUROC of >0.5). This indicates that the count and type of words used by individuals do provide some insights into their anxiety, which is in line with prior work [14,16-19] that explored the association between LIWC features and anxiety.

The performance of the fine-tuned transformer model was larger than the baseline model by 10%—suggesting that it is context aware. We believe that a model that considers context can achieve higher predictive performance. This suggests that transformer models, which search for multiword contexts to find patterns, can extract more information for prediction than single word–based models. The results presented in Tables 2 and 3 allow us to understand, in more detail, what the fine-tuned transformer model based its predictions on, as discussed in the subsequent section.

Furthermore, we note that it is possible to increase the probability of correct prediction by incorporating acoustic features in the prediction of the transformer-based model as well as by using multiple measurements if the circumstances of the measurement system permit it. This would be the case if this kind of a model is applied to passively collected speech, and we could sample the speech and measure it over time. In that case, one could survey the multiple measurements and select the majority result (anxious or nonanxious) that has been predicted as the true result. This approach works under the assumption that each measurement from a different speech sample is independent and will work less well as a function of independence. We have discussed this approach in more detail in our earlier paper [24].

#### Model Interpretation

In this section, we discuss our attempt to provide an interpretation of the results from the transformer model. Table 2 shows the tokens with a high attribution score, as defined earlier, and a high count at influencing the prediction toward anxious and nonanxious. The first entries in Table 3 describe the effects of the singular pronoun “I.” Depending on the context, the use of the word “I” influences *either* toward an anxious prediction or toward a nonanxious prediction. By contrast, previous studies have shown an increased use of “I” to be associated only toward the direction of anxiety [16]. A possible reason why “I” is associated with anxiety is because individuals with anxiety will try to divert their attention from anxiety-inducing events by focusing on themselves. This might result in the frequent use of “I” in their speech.

However, this study shows how the context around the word “I” matters—although its presence influenced the prediction toward anxiety for the majority of the cases (88%), it also influenced the prediction toward nonanxious in 12% of the cases. A pattern around “I” that influenced the prediction toward nonanxious is when it was used to reference others (eg, “I was able to remember all *their* names”). This is opposite to the case where anxious individuals tend to focus on themselves and hence a possible reason as to why focusing on others would influence the prediction toward nonanxious. Another pattern of “I*”* that influenced the prediction toward nonanxious is when it was one of the very first words at the beginning of speech (ie, at the very beginning). This may be because confident people might start their speech by introducing themselves or placing the focus on themselves before proceeding with whatever the subject matter of their speech is. Similarly, relating to confidence, there is a pattern where “I” was used to say something positive about oneself, which influenced the prediction toward nonanxious. These cases suggest that confidence is related to the state of being nonanxious.

Silent pauses ([Silent pause] in Tables 2 and 3) mainly influenced the prediction toward nonanxious, for 80% of the cases. This is in line with prior work [45], which indicated that anxiety is associated with a reduction in the number of silent pauses during speech. The authors suggested that pausing during speech represents a cognitive activity that is observed more in nonanxious individuals than in anxious individuals.

However, there were also times when a silent pause influenced the prediction toward anxiety. The difference was the context: when a silent pause was used together with a filled pause and pausing after saying a single word. These cases hint toward difficulty in producing complete sentences and instead using filler words in the middle of their speech or inability to finish a sentence. This might be because of a higher level of anxiety.

The other 2 types of tokens presented in Table 2 ([Filled Pauses] and “and”) had a high count in influencing the prediction toward both anxious and nonanxious. We believe that they have a high count because they are commonly used tokens in STT transcripts. A pattern that stood out around both ([Filled Pauses] and “and”) types of tokens is the use of grammatically correct language, which was exhibited more by the participants without anxiety. Prior work [46] suggests that anxiety causes disfluencies in speech, which, therefore, could be a possible explanation for the use of grammatically incorrect language by the participants with anxiety. Our results suggest that the model is picking up on this grammatical incorrectness.

### Limitations

One limitation of this study is the accuracy of the STT transcription. In this study, we used Amazon’s STT program [39], which had good transcription accuracy, with an average WER of 7% (SD 4.6%). The fact that the WER is not 0 means that we obtain the wrong transcription for some words, and our model might make a wrong prediction based on these words. However, we speculate that because the STT software is improving each year, the WER would become closer and closer to 0, so the prediction of a model based on these transcripts would also improve.

Another limitation of this study is the use of a modified version of the TSST. In the original TSST, participants are asked to describe why they should be hired for their dream job in front of a live panel of judges. However, in our study, we asked the recruited participants to describe why they should be hired for their dream job in front of a camera at their own location. This is a limitation in achieving the full replication of the TSST as a stress induction task. Nonetheless, we had an internal check where we asked them how anxious they felt before and after the TSST task (more information can be found in our earlier published study [23]), and we observed, on average, a 25% increase in the participants’ level of anxiety.

Another limitation is the use of self-report measures to assess GAD. Self-report measures are subjective opinions that individuals have about themselves and may not completely capture clinical symptoms. Ideally, we would want the gold standard label for determining whether a participant has GAD. This is acquired through a one-on-one session between a patient and clinician where the clinician analyzes the patient’s behavior to identify possible symptoms of GAD according to the Diagnostic and Statistical Manual of Mental Disorders, Fifth Edition [7], but this is clearly much more expensive to acquire.

Another limitation is the subjective or qualitative nature of pattern detection, which is presented in Table 3 and forms the basis of the insights in the *Discussion* section. As described in the *Methods* section, the transcripts were analyzed manually, and instances that we believed exhibited similar patterns across multiple contexts were selected. These were our subjective opinions of what constituted a similar pattern; therefore, other researchers might be able to find other patterns that we might have overlooked. In future studies, we aim to release our transcripts for other researchers to go through as we did and see whether any other interesting patterns could be detected.

### Conclusions

In this paper, we have presented the results of a large-sample study that aimed to predict whether participants who provided speech samples fell below or above the screening threshold for GAD based on the GAD-7 scale. More specifically, we investigated the importance of multiword context when predicting the presence or absence of anxiety. Although prior studies have shown that the choice of individual words is a good predictor of mental health disorders, we have shown that the choice of words together with the context is an even better predictor. Furthermore, transformer-based neural network models can be leveraged to find such linguistic patterns that help identify whether a certain word, given the context, would predict anxiety. There is a type of transformer-based model recently published in the literature [47], which is a model pretrained on a mental health corpus (focusing on depression and suicidality). Therefore, we recommend that future studies explore the linguistic patterns of speech identified using transformer models and apply them to the screening of different types of mental health disorders.

## Abbreviations

AUROC: area under the receiver operating characteristic curve
BIS/BAS: behavioral inhibition/behavioral approach system
GAD: generalized anxiety disorder
GAD-7: Generalized Anxiety Disorder 7-item
IG: integrated gradient
LIWC: Linguistic Inquiry and Word Count
NLP: natural language processing
SAD: social anxiety disorder
STT: speech-to-text
SVM: support vector machine
TSST: Trier Social Stress Test
WER: word error rate
